# Saroglitazar in patients with non-alcoholic fatty liver disease and diabetic dyslipidemia: a prospective, observational, real world study

**DOI:** 10.1038/s41598-020-78342-x

**Published:** 2020-12-03

**Authors:** Omesh Goyal, Sahil Nohria, Prerna Goyal, Jaskirat Kaur, Sarit Sharma, Ajit Sood, Rajoo Singh Chhina

**Affiliations:** 1 Department of Gastroenterology, Dayanand Medical College and Hospital, Ludhiana, Punjab India; 2 Department of Medicine, Dayanand Medical College and Hospital, Ludhiana, Punjab India; 3 Dayanand Medical College and Hospital, Ludhiana, Punjab India

**Keywords:** Diseases, Endocrinology, Gastroenterology, Health care, Medical research

## Abstract

Saroglitazar, a dual peroxisome proliferator activated receptor α/γ agonist, approved for diabetic dyslipidemia (DD), is potential therapeutic option for non-alcoholic fatty liver disease (NAFLD). This prospective, observational, real-world study aimed to determine efficacy and safety of Saroglitazar in patients with NAFLD and DD. We included patients with DD and NAFLD who received Saroglitazar 4 mg once daily for 24 weeks. Blood investigations, liver stiffness measurement (LSM) and controlled attenuation parameter (CAP) (FibroScan) were compared at baseline and 24 weeks. Of 163 patients screened, 107 were included, and 101 completed 24 weeks treatment (mean age 50.4 ± 12.3 years, 78.5% males, mean body mass index 28.8 ± 4.2). After 24 weeks, alanine transaminase (ALT) reduced significantly from 94 (47–122) to 39 (31–49) (*p* < 0.0001) and aspartate aminotransferase (AST) (U/L) from 89 (43–114) to 37 (30–47) (*p* < 0.0001) and LSM (kPa) from 8.4 (7.1–9.3) to 7.5 (6.4–8.4) (*p* = 0.0261). CAP, glycated hemoglobin and lipid parameters also improved significantly. On linear regression, there was significant association between percent change in ALT and AST with TG reduction after treatment (*p* = 0.024 and 0.037 respectively).We conclude that Saroglitazar leads to significant improvement in transaminases, LSM, and CAP in NAFLD patients with DD.

## Introduction

Nonalcoholic fatty liver disease (NAFLD) is one of the most common causes of chronic liver disease in the world^1^. The spectrum of NAFLD ranges from nonalcoholic fatty liver to nonalcoholic steatohepatitis (NASH), which can further progress to cirrhosis and hepatocellular carcinoma. Characterized by presence of insulin resistance (IR), dyslipidemia, and proinflammatory state, NAFLD is often considered as the hepatic component of metabolic syndrome. NAFLD has a strong association with Type 2 Diabetes Mellitus (T2DM), being present in 70%-80% of patients with T2DM. The specific alteration in lipid profile in T2DM patients characterised by increased triglyceride (TG), increased proportion of small dense low-density lipoprotein cholesterol (LDL-C) and decreased high-density lipoprotein cholesterol (HDL-C) is known as diabetic dyslipidemia (DD)^2^. Both NAFLD and T2DM act synergistically. T2DM increases the risk of NASH and hepatocellular carcinoma in NAFLD patients while NAFLD increases the subclinical atherosclerosis increasing risk of complications in T2DM.

The pharmaco-therapeutic options for treatment for NAFLD are limited, and treatment has mainly focused on lifestyle interventions, which are difficult to achieve and sustain by most of the patients^3^. The prime end-point for efficacy of pharmacological interventions against NAFLD should be their impact on liver fibrosis, because the extent of fibrosis has been linked to both hepatic and extrahepatic morbidity and mortality in NAFLD^4^.

**Figure 1:**
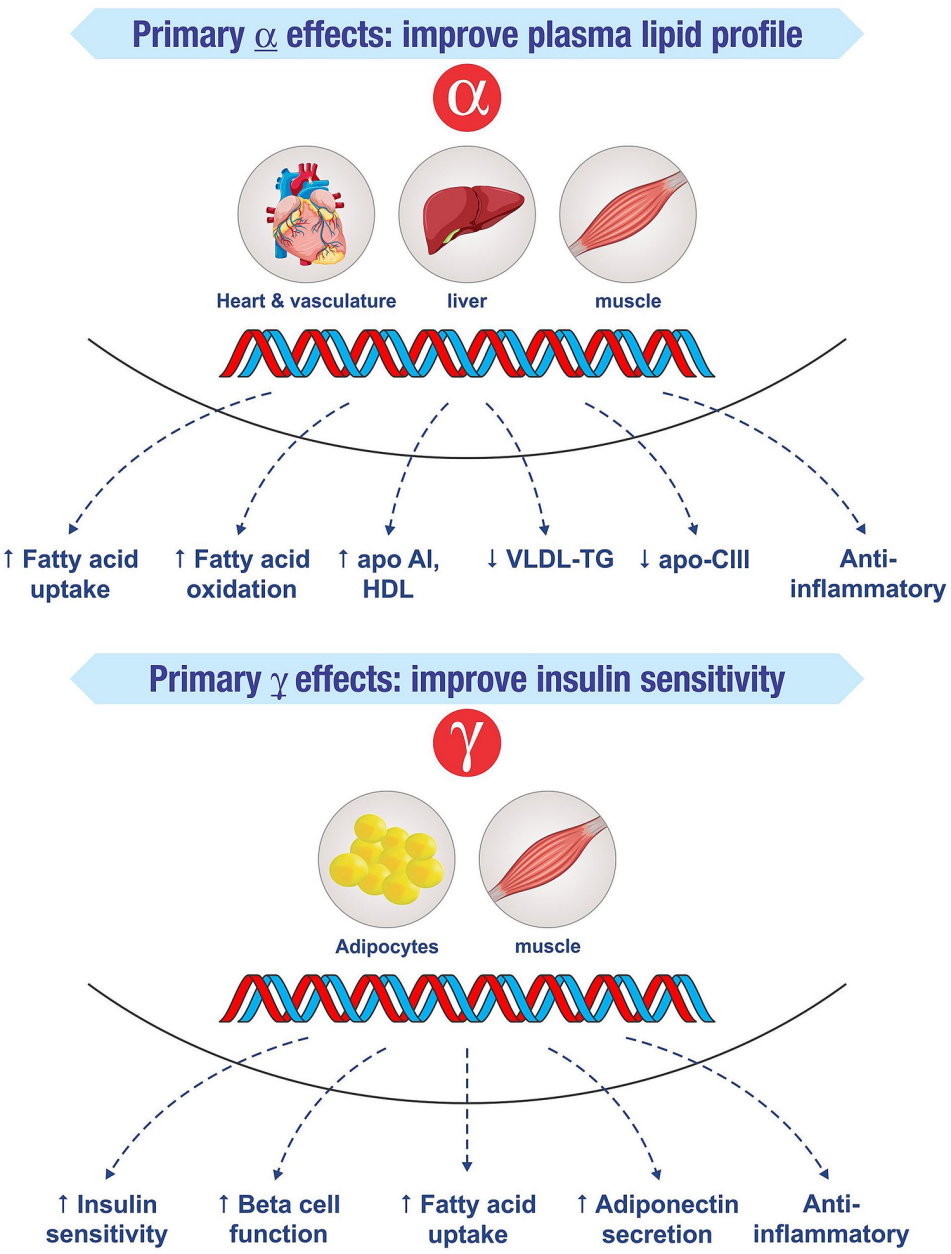
Mechanism of action of Saroglitazar: peroxisome proliferator activated receptor (PPAR) α and γ activation.

Peroxisome proliferator activated receptors (PPARs) are nuclear receptors playing key roles in the regulation of metabolic homoeostasis, inflammation, cellular growth and differentiation. There are mainly three isoforms: alpha (α) present in liver, beta (β)/delta (δ) in skeletal muscle, and gamma (γ) in adipose tissue. Drugs acting on both PPARα and γ (glitazars) address two important issues of NAFLD—dyslipidemia and IR, and thus are the area of interest. Several glitazars (Tesaglitazar, Muraglitazar, Aleglitazar) have been tried in the treatment of DD but their development was terminated because of the adverse events due to their significant γ action. Saroglitazar, a novel dual PPAR ɑ/γ agonist,with predominant PPARα effect and moderate PPARγ effect, lacks these side effects (Fig. 1). Saroglitazar received approval from Drugs Controller General of India for treatment of patients with DD in 2013^5^.

Data on the efficacy of Saroglitazar in NAFLD is emerging. In mice model, beneficial effects of Saroglitazar in NASH were better than pure PPARα agonist, fenofibrate and PPARγ agonist pioglitazone^6,7^. There has been no published human trial documenting the safety and efficacy of Saroglitazar in NAFLD till date. However, data on this subject have been presented at two conferences recently^8,9^. A phase III, multi-center, placebo controlled trial with paired liver biopsies conducted in India by Sarin et al. reported improvement in biochemical parameters and liver biopsy in NASH patients after treatment with 4 mg Saroglitazarfor 52 weeks^8^. A phase II study done in western population reported efficacy of Saroglitazar 4 mg in NAFLD/NASH patients using Magnetic Resonance Proton Density Fat Fraction (MR-PDFF) for categorizing fat content^9^. However, liver biopsy (the current gold standard) and MR-PDFF have limitations of being invasive, costly or lack of easy availability.

Vibration-controlled transient elastography (FibroScan) is a novel, non-invasive technique to assess hepatic fibrosis and steatosis. Shear waves are delivered over right lobe of liver and returning shear wave velocities are measured to generate liver stiffness measurement (LSM) and controlled attenuation parameter (CAP) which correlate with fibrosis and steatosis respectively. Transient elastography (TE) has advantages of being quick, non-invasive, well tolerated, and covering 100 times bigger volume of liver than liver biopsy^10^. TE has been reported to be accurate and reliable non-invasive method to assess liver fibrosis and steatosis in patients with NAFLD^11–15^.

There is lack of prospective clinical data on the effect of Saroglitazar on transaminases, liver fibrosis and liver steatosis assessed by non-invasive methods like FibroScan in patients with NAFLD. Hence, this study was conducted to determine the efficacy and safety of Saroglitazar in NAFLD patients with DD in a real-world setting.

## Materials and methods

### Study population

This prospective, observational, single-arm, real world study was conducted in a tertiary care institute in northern India from May 2019 to April 2020. Study protocol was approved by institutional ethics committee and was prospectively registered with CTRI (CTRI/2019/05/019199). Study was conducted in accordance with the ethical standards of the Helsinki Declaration of 1975, as revised in 2008. Informed consent was taken from all subjects.

Patients with DD who were prescribed Saroglitazar 4 mg once daily by the treating physician as per standard of care in the outpatient department were enrolled. *Inclusion criteria* were—age 18–70 years; presence of diabetes with dyslipidemia (HbA1c > 6.2%, total cholesterol > 200 mg/dl, triglycerides > 150 mg/dl) and evidence of fatty liver on ultrasound. *Exclusion criteria* were—chronic hepatitis B or C, significant alcohol intake (> 210 g/week in males and > 140 g/week in females), use of thiazolidinediones or Saroglitazar in past 6 months, use of drugs associated with hepatotoxicity/hepatic fibrosis (amiodarone, anabolic steroids, antiretroviral drugs, chloroquine, estrogens, high dose vitamin A, methotrexate, oral contraceptives, sodium valproate, systemic glucocorticoids, tamoxifen, tetracycline etc.), liver diseases due to other etiologies like autoimmune liver disease, Wilsons’ disease etc., cirrhosis on ultrasound or TE value > 11.5 kPa^11^, uncontrolled thyroid disease, active cardio-pulmonary disease or chronic kidney disease. Patients were prospectively followed up every 3–4 weeks either on their scheduled out-patient department visits or telephonically. Primary outcome was the effect of Saroglitazar on transaminases, and secondary outcome was effect of Saroglitazar on LSM and CAP.

### Measurements and analytical determinations

Baseline investigations including fasting blood sugar, liver function tests, fasting lipid profile, glycated hemoglobin (HbA1c), creatinine, hemogram, ultrasound abdomen, FibroScan were noted. Patients who completed at least 24 weeks of therapy with Saroglitazar, and got repeat blood investigations during 20–30 week of follow up were included for final analysis. Some patients did not get FibroScan at baseline/follow-up (due to financial reasons) but were included in the study, as LSM/CAP assessment was a secondary outcome. Compliance to treatment was assessed by checking empty containers of the drug.

All pre- and post-treatment investigations were done at the institute’s laboratory. Ultrasound was done by a senior radiologist. Grades of fatty liver were defined as: Grade I—echogenicity just increased; Grade II—echogenic liver obscures echogenic walls of portal vein branches, and Grade III—echogenic liver obscures diaphragmatic outline. TE with FibroScan (Echosens; Paris, France) was used to measure the LSM and CAP values, using M or XL probes, as per manufacturers instructions. Procedure was performed after an overnight fast, by a senior operator with an experience of performing > 5000 FibroScan procedures. LSM was performed on right lobe of liver, with patient in supine position. Ten successful acquisitions were performed on each patient. Results of LSM and CAP were expressed as median (M) and interquartile range (IQR) of all valid measurements. IQR was defined as value corresponding to interval containing 50% of valid measurements between the 25^th^ and 75^th^ percentiles. As an indicator of intrinsic variability, ratios of IQR of LSM and CAP values to their median values (IQR/M) were calculated. Results were considered reliable only if IQR/M was ≤ 0.30. Operator was blind to all the clinical data. Procedure failure was defined as failure to obtain any valid measurement. LSM and CAP cut-offs were taken as reported previously^11,12^. Significant fibrosis ≥ F2 was taken as LSM > 7.0 kPa.

### Statistical analysis

The data were checked for normal distribution using Shapiro Wilk test. Categorical data are presented as proportions, and continuous data are presented as mean and standard deviation (if parametric) and median and inter-quartile range (if non-parametric). An intention to treat analysis was performed. Multiple ANOVA or Friedman tests were used, where appropriate, to compare pre- and post-treatment data. Multiple linear regression analysis was performed with percent change in ALT and AST after treatment as dependent variables, and percent changes in BMI, HbA1c, TG, non-HDL-C, LSM and CAP as independent variables to determine potential association between these factors. Correlations between baseline alanine transaminase (ALT), LSM and CAP were determined by Spearman’s correlation coefficient. *p* values < 0.05 were considered significant. Statistical analysis was done using Epi-Info and statistical package of social sciences (SPSS) version 21 (SPSS, Inc., Chicago, IL, USA).

### Ethics standards

All procedures followed were in accordance with the ethical standards of the responsible committee on human experimentation (institutional and national) and with the Helsinki Declaration of 1975, as revised in 2008.

## Results

**Figure 2:**
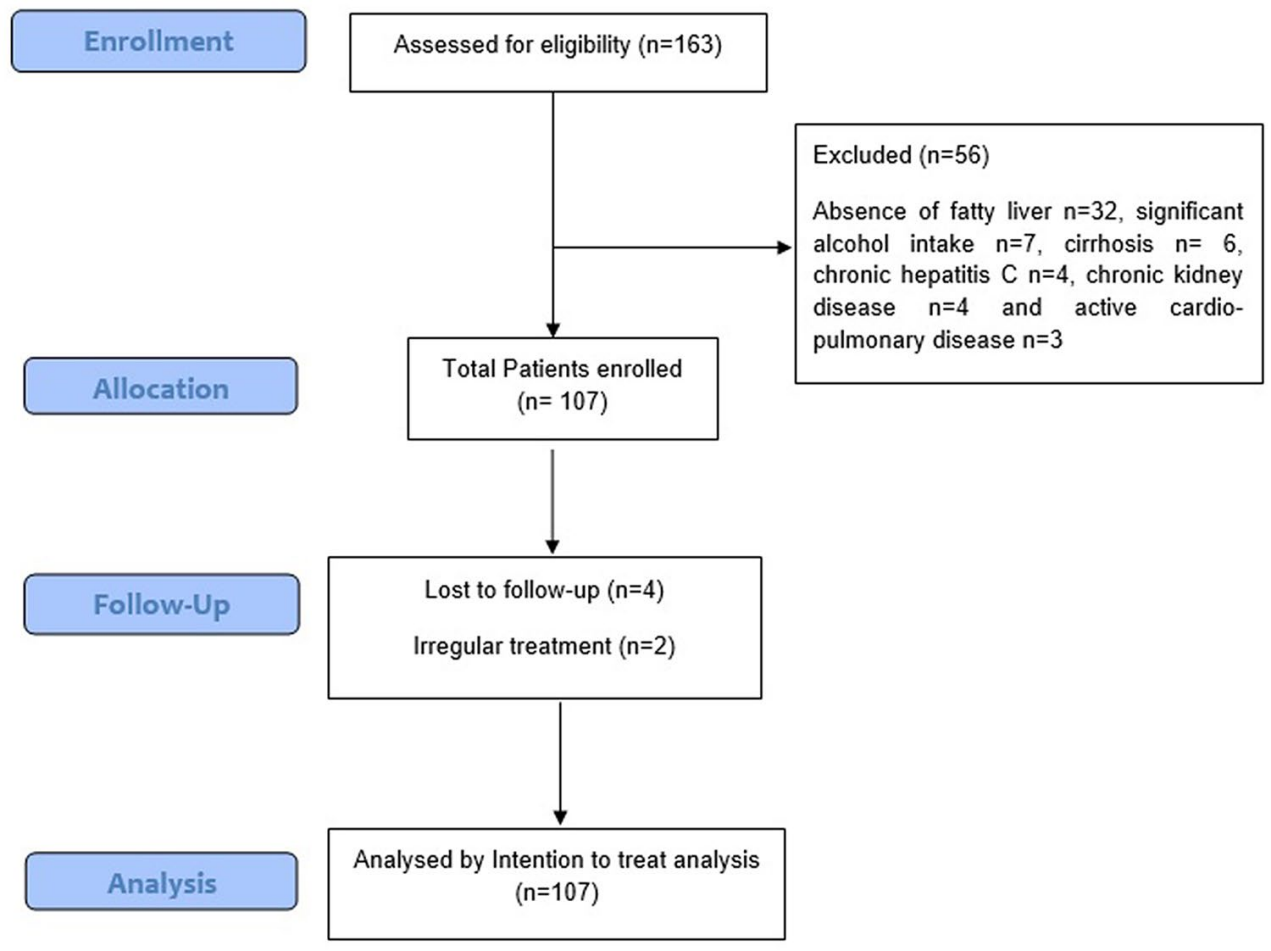
Flow of participants in the study.

Total of 163 patients with diabetes and dyslipidemia were screened, out of whom 107 were included (Fig. 2). Out of these, 6 were lost to follow up or took irregular treatment, and 101 patients completed 24 weeks of treatment with Saroglitazar. Biochemical investigations at baseline and 24 weeks were available for all the patients. However, FibroScan was available for 91 patients at baseline (8 patients did not get FibroScan; 2 did not have valid FibroScan measurements) and 85 patients at 24 weeks (6 patients did not get repeat FibroScan).

Basic demographic profile of patients is shown in Table 1. The mean age was 50.4 ± 12.3 years, 78.5% were males and mean body mass index (BMI) was 28.8 ± 4.2. The treatment being taken by patients for dyslipidemia, diabetes and hypertension is shown in Table 2. Patients were asked to continue all previous medications except fibrates.

**Table 1:** Demographic profile of patients (n = 107).

**Characteristic**	
Age (years)	50.4 ± 12.3
Male gender	84 (78.5)
Urban residence	71 (66.4)
Weight (kg)	83.5 ± 15.1
BMI (kg/m^2^)	28.8 ± 4.2
Normal BMI (18.5–22.9)	11 (10.3)
Over weight (23–24.9)	21 (19.6)
Pre-obese (25.0–29.9)	37 (34.6)
Obese type 1 (30–39.9)	35 (32.7)
Obese type 2 (40–49.9)	3 (2.8)
Obese type 3 (> 50)	0 (0.0)
SBP (mmHg)	143.5 ± 30.6
DBP (mmHg)	91.4 ± 15.9
**Comorbidities**	
Diabetes	107 (100)
Duration of diabetes (years)	8.4 ± 5.3
Hypertension	56 (52.3)
Coronary artery disease	19 (17.8)
Hypothyroidism	11 (10.3)

Data are expressed as mean ± S.D or number (percentage).

BMI, Body mass index; DBP, Diastolic Blood pressure; SBP, Systolic Blood Pressure.

**Table 2:** Pre-enrollment medication details of patients (n = 107).

Drugs	Number (percent)
**Drugs for Dyslipidemia**	
Atorvastatin	29 (27.1)
Rosuvastatin	16 (15.0)
Simvastatin	6 (5.6)
Fenofibrates	11 (10.3)
Statin plus fibrates	27 (25.2)
Statin plus Ezetamide	7 (6.5)
No treatment	11 (10.3)
**Anti-diabetic drugs**	
Metformin	94 (87.9)
Sufonlyureas	51 (47.7)
Gliptins	26 (24.3)
Alpha glucosidase inhibitors	19 (17.8)
SGLT 2 inhibitors	15 (14.0)
Insulin	15 (14.0)
No treatment	9 (8.4)
**Anti-hypertensives**	
ACEI/ARBs	23 (21.5)
CCB	14 (13.1)
Diuretics	15 (14.0)
Beta blockers	12 (11.2)
Others	10 (9.3)

ACEI, Angiotensin Converting Enzyme Inhibitor; ARBs, Angiotensin Receptor Blocker; CCB, Calcium channel Blocker; SGLT, Sodium Glucose Linked Transporter.

### Baseline investigations

Baseline investigations are shown in Table 3. The median ALT and aspartate aminotransferase (AST) values were 94 (47–122) and 89 (43–114) IU/L respectively. ALT and AST values were greater than 40 IU/L in 78.5% (n = 84) and 75.7% (n = 81) patients respectively. Serum albumin was in normal range in all patients, indirect bilirubin was high in three patients and ALP was increased to < 2 times upper limit of normal in 17.7% (n = 19) patients. The success rate of FibroScan was 97.8% (89/91). The median LSM value was 8.4 (7.1–9.3) kPa and the median CAP value was 335 (281–392) dB/m. Total 72.5% (66/91) patients had LSM value ≥ 7.0 kPa (suggestive of ≥ F2 fibrosis). Out of these, 9.1% (6/66) patients had normal ALT. Detailed distribution of LSM values, CAP values and fatty liver grades on ultrasound are shown in Table 4.

**Table 3:** Comparison of baseline and post treatment parameters (n = 107).

Parameter	Baseline	24 weeks	*p* value
Bilirubin (mg/dL)	0.78 ± 0.31	0.82 ± 0.37	0.35
ALT (U/L)	94 (47–122)	39 (31–49)	< 0.0001
AST(U/L)	89 (43–114)	37 (30–47)	< 0.0001
ALP (U/L)	91 (72–142)	88 (76–139)	0.731
Albumin (g/dL)	4.6 ± 0.4	4.6 ± 0.5	1.00
Total cholesterol (mg/dL)	209.8 ± 62.4	185.7 ± 22.5	0.0003
LDL-C (mg/dL)	120.9 ± 41.2	106.2 ± 38.2	0.008
HDL-C (mg/dL)	38.2 ± 8.1	43.2 ± 9.8	0.0001
VLDL-C (mg/dL)	50.9 ± 8.5	37.8 ± 9.6	0.007
Triglycerides (mg/dL)	326.4 ± 98.5	168.3 ± 79.7	< 0.0001
non-HDL-C (mg/dL)	169.5 ± 44.6	141.7 ± 37.9	< 0.0001
LDL/HDL Ratio (mg/dL)	3.15 ± 1.1	2.46 ± 0.9	< 0.0001
HbA1c (%)	7.2 ± 0.65	6.3 ± 0.87	0.0001
LSM (kPa)^†^	8.4 (7.1–9.3)	7.5 (6.4–8.4)	0.0261
IQR/M	0.25 (0.19–0.31)	0.24 (0.17–0.30)	0.0067
CAP (dB/m)^†^	335 (281–392)	256 (212–299)	0.0076
IQR/M	0.26 (0.21–0.31)	0.28 (0.23–0.32)	0.0001
Hemoglobin (gm/dL)	14.2 ± 2.3	14.1 ± 2.5	0.76
TLC (10^3^/µl)	6.7 ± 2.9	7.4 ± 3.1	0.09
Platelets (10^3^/µl)	194 ± 64	187 ± 51	0.38
Creatinine (mg/dl)	0.92 ± 0.3	0.94 ± 0.3	0.63
SBP (mm Hg)	143.5 ± 30.6	141.6 ± 28.7	0.64
DBP (mm Hg)	91.4 ± 15.9	93.1 ± 16.9	0.44
Weight (kg)	83.5 ± 15.1	80.4 ± 11.5	0.09
BMI (kg/m^2^)	28.8 ± 4.2	27.1 ± 6.3	0.06

Data are expressed as mean ± S.D or median (inter-quartile range).

ALP, Alkaline Phosphatase; ALT, Alanine transaminase; AST, Aspartate aminotransferase; CAP, Controlled Attenuation parameter; DBP, Diastolic Blood pressure; HbA1c, Glycated Hemoglobin; HDL-C, High Density lipoprotein cholesterol; IQR/M, Interquartile Range/median; LDL-C, Low Density lipoprotein cholesterol; LSM, Liver stiffness measurement; SBP, Systolic Blood Pressure; TLC, Total leukocyte count; VLDL-C, Very Low Density lipoprotein cholesterol.

^†^n = 91 at baseline and n = 85 at 24 weeks.

**Table 4:** Results of Ultrasound, Liver stiffness measurement (LSM) and Controlled Attenuation parameter (CAP) at baseline (n = 107).

Characteristic	Number (percent)
**Ultrasound grades of Fatty Liver**	
Grade 1	24 (22.43)
Grade II	66 (61.68)
Grade III	17 (15.88)
**Liver stiffness measurement (LSM), kPa**^†^	
< 7.0 (F1)	25 (27.10)
7.0–8.6 (F2)	38 (42.05)
8.7–11.5 (F3)	28 (30.84)
**Controlled Attenuation parameter (CAP), dB/m**^†^	
223–310 (S1)	14 (14.95)
311–339 (S2)	48 (53.27)
> 340 (S3)	29 (31.77)

^†^n = 91.

### Post-treatment investigations

**Figure 3:**
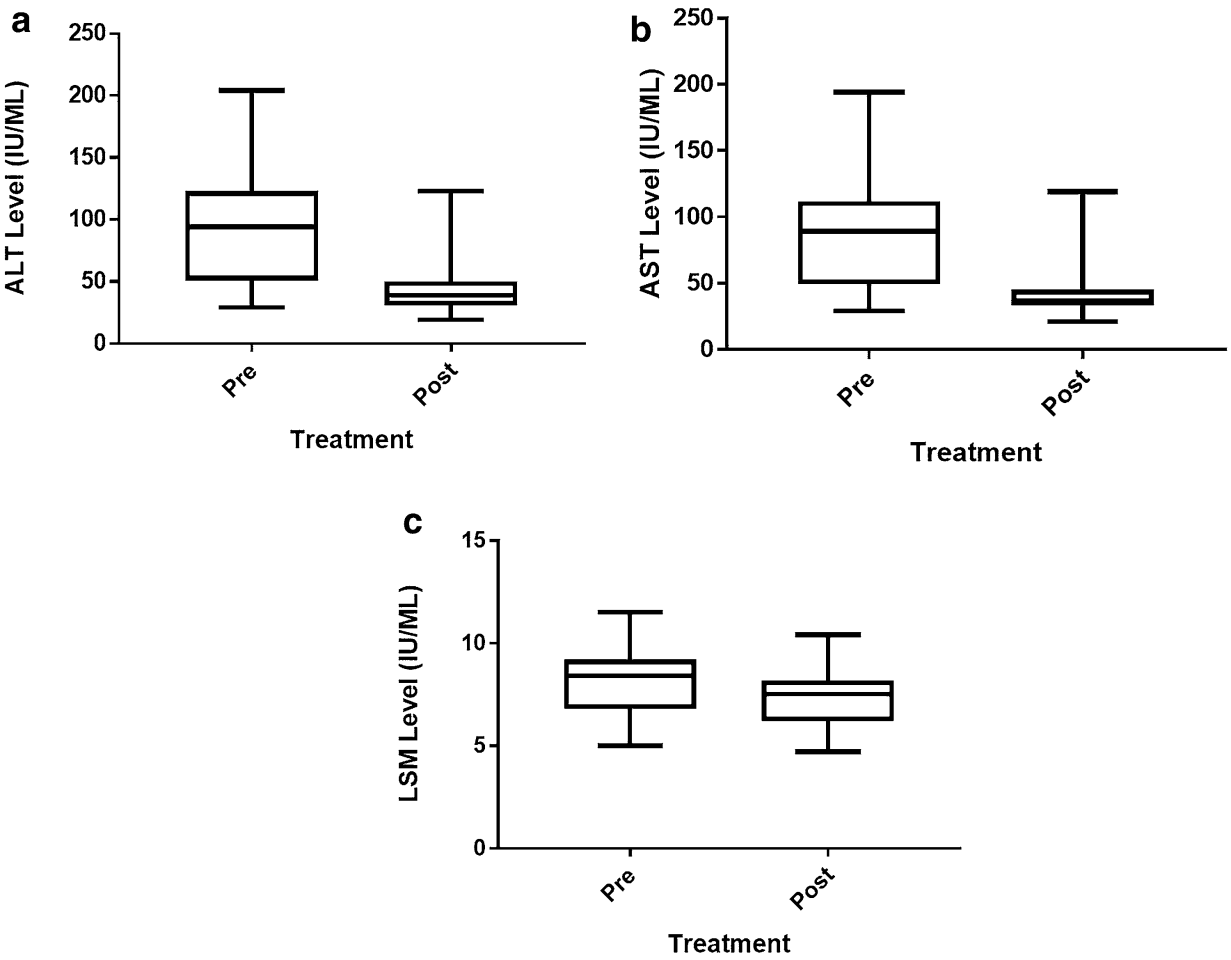
Changes in various parameters after 24 weeks of Saroglitazar treatment (**a**) Alanine transaminase (ALT), (**b**) Aspartate aminotransferase (AST), and (**c**) Liver stiffness measurement (LSM).

After 24 weeks therapy with Saroglitazar, the median ALT (U/L) reduced to 39 (31–49) (*p* < 0.0001) and median AST (U/L) to 37 (30–47) (*p* < 0.0001) (Table 3, Fig. 3). Sixty one (60.4%) patients had ALT values ≤ 40, and 66 (65.4%) patients had AST values ≤ 40. Also, LSM, CAP, lipid profile and HbA1c showed significant reduction, while there was no significant change in serum bilirubin, alkaline phosphatase, protein, albumin levels, weight or BMI **(**Table 3). A post-hoc analysis including patients with F2/F3 fibrosis (n = 66) was conducted to assess change in LSM after treatment. In this subgroup, LSM reduced from 8.7 (8.3–9.5) to 7.9 (7.4–8.7) kPa (*p* < 0.0001).

Saroglitazar was well tolerated. Minor adverse events reported were—fatigue in 2.8% (n = 3), nausea in 1.9% (n = 2) and dyspepsia 1.9% (n = 2) which were managed symptomatically.

### Correlation and regression analysis

At baseline, ALT values had no significant correlation with LSM (r = 0.35, *p* = 0.094) or CAP (r = 0.21, *p* = 0.821). LSM and CAP values showed a moderate positive correlation (r = 0.57, *p* = 0.079), however, it was not statistically significant. Multiple linear regression analysis showed a significant association between percent change in ALT and AST with percent change in TG after treatment (r = 0.713; *p* = 0.024 and and r = 0.648; *p* = 0.037 respectively). There was no significant association between ALT or AST reduction with percent change in non-HDL cholesterol (r = 0.318; *p* = 0.085), BMI (r = 0.231; *p* = 0.434), HbA1c (r = 0.087, *p* = 0.782), LSM (r = 0.298, *p* = 0.091) or CAP (r = 0.446, *p* = 0.078).

## Discussion

The present study reports the efficacy of Saroglitazar in improving transaminases, dyslipidemia and glycemic control in NAFLD patients with DD in a real world setting. It is probably the first study to document the effect of Saroglitazar on liver fibrosis (LSM) and steatosis (CAP) assessed by non-invasive method i.e. TE (FibroScan).

Various drugs have been evaluated for the treatment of NAFLD. PPARγ agonists (Pioglitazone, rosilglitazone) showed some benefit with their antifibrotic properties but didn’t gain widespread use because of their side effects like risk of bladder cancer, cardiac events and weight gain^5^. Fibrates, weak PPARα agonists, showed limited efficacy in NASH^5^, while Elafibranor, PPAR α and δ agonist, has been shown to improve NASH parameters^16^. Vitamin E, metformin, and silymarin haven’t proved effective for management of NASH^17^. Recently, Obeticholic acid and Liraglutide have shown some promising results in treatment of NAFLD but more data on their efficacy and safety is required before these drugs get approval for NAFLD treatment^18,19^. Saroglitazar, a dual α/γ agonist, has a well-established role in the management of DD^20–23^. Jain et al. reported that Saroglitazar reduces hypertriglyceridemia and improves insulin sensitivity along with β-cell function by reduction in gluco-lipotoxicity and possibly directly through PPAR-γ agonism in patients of T2DM with hypertriglyceridemia^24^. Recently, Saroglitazar has stimulated interest of physicians for treatment of NAFLD due to its dual effect in improving dyslipidemia and insulin sensitivity.

Elevated transaminases are a marker of ongoing hepatocyte injury and commonly deranged in patients with NAFLD. Although transminases do not correlate with hepatic fibrosis^25^, regular monitoring and follow-up of transaminase levels in NAFLD patients is commonly used in routine practise as well as clinical drug trials because of the ubiquitous availability and low cost. There is limited data on effect of Saroglitazar treatment on improvement in transaminases. Two recent studies available in abstract form have reported improvement in liver biochemistry with Saroglitazar in NAFLD^8,9^. In the EVIDENCE IV trial, greatest decrease in ALT value was observed in group which received 4 mg Saroglitazar compared to the group who received 2 mg and 1 mg^9^. Another study published in abstract form only reported reduction in transaminases with Saroglitazar^26^. In the present study, there was significant reduction in mean ALT and AST after 24 weeks of Saroglitazar treatment.

Hepatic fibrosis is the ideal end-point for assessing efficacy of pharmacological interventions for NAFLD^4^, and liver biopsy is considered the gold standard to assess liver fibrosis. However, data on the accuracy of LSM to assess liver fibrosis in NAFLD patients is emerging^10–15^. Two studies reported utility of LSM to assess changes in liver fibrosis after bariatric surgery^27,28^. In our study, there was significant improvement in LSM values after 24 weeks of Saroglitazar treatment, and this improvement was noted even in the sub-group with F2/F3 fibrosis. In a recent biopsy study by Sarin et al., there was improvement in necro-inflammation with no change in fibrosis after Saroglitazar treatment^8^. This difference could be due to the difference in baseline population. While our study included NAFLD patients with diabetes and dyslipidemia, Sarin et al. included all NAFLD patients without this restriction. Sub-group analysis of the latter study would be helpful to clarify this point. The other possibility of LSM values being influenced by steatosis or inflammation is still debatable^11,29^. Future long-term studies performing both liver biopsy and TE in NAFLD patients are needed to confirm the effect of Saroglitazar on liver fibrosis in all subgroups of NAFLD patients.

Assessment of change in hepatic steatosis is another important aspect of assessing response to therapy in NAFLD. Although abdominal ultrasonography is often the first-line investigation for diagnosis of fatty liver, it is operator-dependent and falsely negative when < 30% of hepatocytes are steatotic. CAP assessment is a reasonable test for assessment of steatosis in NAFLD. A recent study reported good accuracy of CAP in assessing change in hepatic steatosis after bariatric surgery in NAFLD patients^27^. MR-PDFF is another technique to quantitatively assess liver fat. In a recent study, there was > 30% reduction in liver fat content measured by MR-PDFF, after treatment with Saroglitazar^9^. Although MRI-PDFF has shown to be superior than CAP, the former is limited by cost and availability^30^. In concordance with above study, we observed significant reduction in CAP values after 24 weeks of Saroglitazar treatment, suggesting improvement in hepatic steatosis. Sarin et al. also reported improvement in steatosis and inflammation on liver biopsy with Saroglitazar treatment^8^.

As expected, a favourable effect of Saroglitazar on lipid profile was noted in our study. After 24 weeks of treatment, serum TG significantly reduced from 326.4 ± 98.5 mg/dL to 168.3 ± 79.7 mg/dL. Other parameters like LDL-C, VLDL-C, total cholesterol and HDL-C also showed significant improvement. Our results are consistent with other observational studies of Saroglitazar in DD. Shetty et al. reported significant decrease in TG after 12 weeks treatment (312.3 ± 122.7 mg/ dL to 188.7 ± 61.4 mg/dL)^20^. In the GLIDDER study, there was similar improvement in lipid profile^21^. In PRESS V study, Saroglitazar 4 mg significantly reduced plasma TG from baseline by 45% at week 24^31^. In PRESS VI study, Saroglitazar showed significant improvement in TG over 12 weeks, in patients in whom Atorvastatin therapy was not effective^32^. Recently conducted phase II and III trials of Saroglitazar in NAFLD, also reported significant improvement in lipid paramenters with 4 mg Saroglitazar^8,9^. HbA1c is used to assess the glycemic control over past 12 weeks. HbA1c is involved in pathogenesis of NAFLD through various pathways, so improvement in HbA1c also has positive impact on NAFLD^33^. Saroglitazar has been reported to provide significant reduction in HbA1c in DD patients^20–24^. In our study too, we observed a significant reduction in HbA1c with Saroglitazar.

Minor adverse events like dyspepsia and fatigue were reported in our study, but none required treatment discontinuation. PRESS V study reported gastritis, tremors and giddiness with Saroglitazar^31^.

Our study has few limitations. The study included patients of NAFLD who had DD, as Saroglitazar had been approved to be used in this group of patients only, when this study was conducted. So, our results cannot be generalised to NAFLD patients without DD. Secondly, this study was not a randomised, placebo controlled trial, and there was no control group. However, as the efficacy assessment parameters (liver biochemistry, FibroScan) were all objective, any type of bias is unlikely to affect the results. Not conducting a liver biopsy could be considered another limitation, but as this study was an observational study in the real world setting, performing a liver biopsy was not possible, as it is rarely done for assessing fibrosis/steatosis in NAFLD patients in the realworld scenario, especially with the availability of good non-invasive tests like TE (FibroScan). The use of other concomitant anti-diabetic drugs in our study is unlikely to affect the results, as the patients enrolled in this study were already on these drugs since past many months, and these were continued in the same doses during the study period. Moreover, there is no convincing data that existing anti-diabetic drugs are effective in improving fibrosis/steatosis in NASH.

The strength of the study is that it is the first prospective, registered, real world study evaluating the efficacy of Saroglitazar in a large cohort of patients with NAFLD and DD. It is also probably the first study which used two non-invasive parameters i.e. LSM and CAP to evaluate the efficacy of a drug on hepatic fibrosis and steatosis in NAFLD patients. A linear regression analysis assessing factors associated with ALT and AST reduction has been performed.

To conclude, Saroglitazar leads to significant improvement in transaminases, LSM, CAP, glycemic control and lipid parameters in NAFLD patients with DD. Therefore, Saroglitazar could be a potentially good therapeutic option fulfilling the unmet need for treatment of NAFLD.

## Data Availability

The datasets generated during and/or analysed during the current study are available from the corresponding author on reasonable request.
